# Supplementary material for: More accurate quantification of model-to-model agreement in externally forced climatic responses over the coming century
Source: Nat Commun. 2021 Feb 4;12:788. doi: 10.1038/s41467-020-20635-w (PMC7862648; doi:10.1038/s41467-020-20635-w)
Supplement: Supplementary file 1 — Supplementary Information [file 41467_2020_20635_MOESM1_ESM.pdf]

Supplementary Information: More accurate quantification of model-to-model agreement in externally forced climatic responses over the coming century

*Nicola Maher[1], Scott B. Power[2,3], and Jochem Marotzke[1]

[1]Max Planck Institute for Meteorology, Hamburg, Germany

[2]Bureau of Meteorology, Melbourne, Victoria, Australia

[3]ARC Centre of Excellence for Climate Extremes, School of Earth, Atmosphere and Environment, Monash University, Melbourne, Australia

**nicola.maher@mpimet.mpg.de*

5 November 2020

Supplementary Information

Supplementary Table 1: The six single model initial-condition large ensembles (SMILEs) used in this paper [1]

SMILE name	ensembles	reference
MPI-GE	100	[2]
CESM-LE	40	[3]
CanESM2	50	[4]
GFDL-ESM2M	30	[5]
GFDL-CM3	20	[6]
CSIRO-Mk3-6-0	30	[7]

Supplementary Table 2: CMIP5 models used in this study and the number of ensemble members.

Modelling Centre	Models	ensembles	r1i1p1
CSIRO-BOM	ACCESS1-0	1	Y
CSIRO-BOM	ACCESS1-3	1	Y
BCC	bcc-csm1-1	1	Y
BCC	bcc-csm1-1-m	1	Y
CCCma	CanESM2	5	Y
NCAR	CCSM4	6	Y
NCAR	CESM1-BGC	1	Y
NSF-DOE-NCAR	CESM1-CAM5-1-FV2	1	Y
NSF-DOE-NCAR	CESM1-CAM5	3	Y
CMCC	CMCC-CESM	1	Y
CMCC	CMCC-CM	1	Y
CMCC	CMCC-CMS	1	Y
CNRM-CERFACS	CNRM-CM5	5	Y
CSIRO-QCCCE	CSIRO-Mk3-6-0	10	Y
ICHEC	EC-EARTH	1	N
IAP	FGOALS-g2	1	Y
FIO	FIO-ESM	3	Y
GFDL	GFDL-CM3	1	Y
GFDL	GFDL-ESM2G	1	Y
GFDL	GFDL-ESM2M	1	Y
GISS	GISS-E2-H-CC	1	Y
GISS	GISS-E2-H	5	Y
GISS	GISS-E2-R-CC	1	Y
GISS	GISS-E2-R	5	Y
MOCH	HadGEM2-ES	4	Y
NIMR	HadGEM2-AO	1	Y
INM	inmcm4	1	Y
IPSL	IPSL-CM5A-LR	4	Y
IPSL	IPSL-CM5A-MR	1	Y
IPSL	IPSL-CM5B-LR	1	Y
MIROC	MIROC5	3	Y
MIROC	MIROC-ESM-CHEM	1	Y
MIRCO	MIROC-ESM	1	Y
MPI	MPI-ESM-LR	3	Y
MPI	MPI-ESM-MR	1	Y
MRI	MRI-CGCM3	1	Y
MRI	MRI-ESM1	1	Y
NCC	NorESM1-M	1	Y

Supplementary Table 3: CMIP5 models grouped into sub-ensembles which share the atmosphere component. The number of ensemble members for each sub-ensemble is shown in brackets next to the group number. The sub-ensembles are created using information from Boé [8].

Group	Models
1 (7)	ACCESS1-0
	ACCESS1-3
	HadGEM2-AO
	HadGEM2-ES
2 (2)	bcc-csm1-1-m
	bcc-csm1-1
3 (15)	FIO-ESM
	CCSM4
	CESM1-BGC
	CESM1-CAM5-1-FV2
	CESM1-CAM5
	NorESM1-M
4 (5)	CanESM2
5 (7)	CMCC-CESM
	CMCC-CMS
	CMCC-CM
	MPI-ESM-LR
	MPI-ESM-MR
6 (10)	CSIRO-Mk3-6-0
7 (1)	EC-EARTH
8 (3)	GFDL-CM3
	GFDL-ESM2G
	GFDL-ESM2M
9 (12)	GISS-E2-H-CC
	GISS-E2-H
	GISS-E2-R-CC
	GISS-E2-R
10 (1)	inmcm4
11 (6)	IPSL-CM5A-LR
	IPSL-CM5A-MR
	IPSL-CM5B-LR
12 (5)	MIROC-ESM-CHEM
	MIROC-ESM
	MIROC5
13 (2)	MRI-CGCM3
	MRI-ESM1
14 (5)	CNRM-CM5

Supplementary Table 4: CMIP5 models grouped into sub-ensembles which share the ocean component. The number of ensemble members for each sub-ensemble is shown in brackets next to the group number. The sub-ensembles are created using information from Boé [8].

Group	Models
1 (5)	HadGEM2-AO
	HadGEM2-ES
2 (16)	bcc-cesm1-1-m
	bcc-csm1-1
	CSIRO-Mk3-6-0
	ACCESS1-0
	ACCESS1-3
	GFDL-CM3
	GFDL-ESM2M
3 (6)	GISS-E2-R-CC
	GISS-E2-R
4 (14)	CCSM4
	CESM1-BGC
	CESM1-CAM5-1-FV2
	CESM1-CAM5
	FIO-ESM
5 (6)	GISS-E2-H-CC
	GISS-E2-H
6 (15)	CMCC-CESM
	CMCC-CMS
	CMCC-CM
	CNRM-CM5
	EC-EARTH
	IPSL-CM5A-LR
	IPSL-CM5A-MR
	IPSL-CM5B-LR
7 (5)	CanESM2
8 (1)	inmcm4
9 (5)	MIROC-ESM-CHEM
	MIROC-ESM
	MIROC5
10 (1)	FGOALS-g2
11 (4)	MPI-ESM-LR
	MPI-ESM-MR
12 (3)	MRI-CGCM3
	MRI-ESM1
	NorESM1-M
13 (1)	GFDL-ESM2G

Supplementary Note 1: Uncertainty due to ensemble size To investigate the uncertainties due to the differing ensemble sizes of the SMILEs we use the largest ensemble (MPI-GE; 100 members) and investigate the range of results that could be obtained by sub-sampling the ensemble for smaller sizes. To do this we investigate the forced response ($\Delta T_{s,\mathrm{F}}$) and the internal variability of the forced response ($\sigma(\Delta T_s)$), estimated for both T and T_σ. We first re-sample MPI-GE 100 times for ensemble sizes of 10, 20, 30, 50 and 75. We complete the re-sampling without replacement, however a test shows that re-sampling with replacement gives the same result (not shown). We then plot the 100-member estimate and the difference between the maximum and minimum result that could be obtained at each ensemble size as a percentage of the 100-member estimate for T (forced response; Supplementary Figure 1, internal variability; Supplementary Figure 2) and T_σ (forced response; Supplementary Figure 3, internal variability; Supplementary Figure 4). We find that for the forced response in T the differences between the maximum and minimum estimate are already fairly low with an ensemble of 10-members, showing that the ensemble size does not introduce large uncertainty in this case, although there are some larger differences in the Southern Ocean and North Atlantic that reduce with increasing ensemble size. For the internal variability of the forced response in T the percentage error is a similar size all around the globe. Here, we see that the uncertainty reduces with increasing ensemble size, but that there is still some uncertainty at even 75 members. For the forced response in T_σ the error reduces as the ensemble size increases, however it is still large at 75 members (note the change in colourbar from 0-200% for this Figure). The large magnitude of the error in this case is likely because $\Delta T_{s,\mathrm{F}}$ is relatively small compared to its mean value. For the internal variability of the forced response in T_σ, the result is very similar to the internal variability of the forced response in T. Again we find that while the error greatly reduces as the ensemble size increases, there is still error at 75 members. This analysis has shown that the ensemble size does indeed introduce uncertainty into our analysis, with larger ensembles giving a much more robust result. In all cases the uncertainty in the 20-member ensemble is reduced compared to the 10-member ensemble, however the 30-member ensemble again reduces the uncertainty as compared to the 20-member ensemble. We choose to keep the 20-member SMILE GFDL-CM3 in our analysis because having the extra data when only six SMILEs are available adds to our analysis of model-to-model agreement, but note that there is error in its estimates.

In the final panel of Supplementary Figures 1, 2, 3, 4 we plot the average of the 100 10-member ensembles subtracted from the 100-member estimate. The minimal differences shown indicate that the quantities plotted are not a function of ensemble size, but a model quantity. I.e. the estimate of internal variability does not increase with increasing ensemble size, and larger ensembles do not necessarily have a larger internal variability estimate.

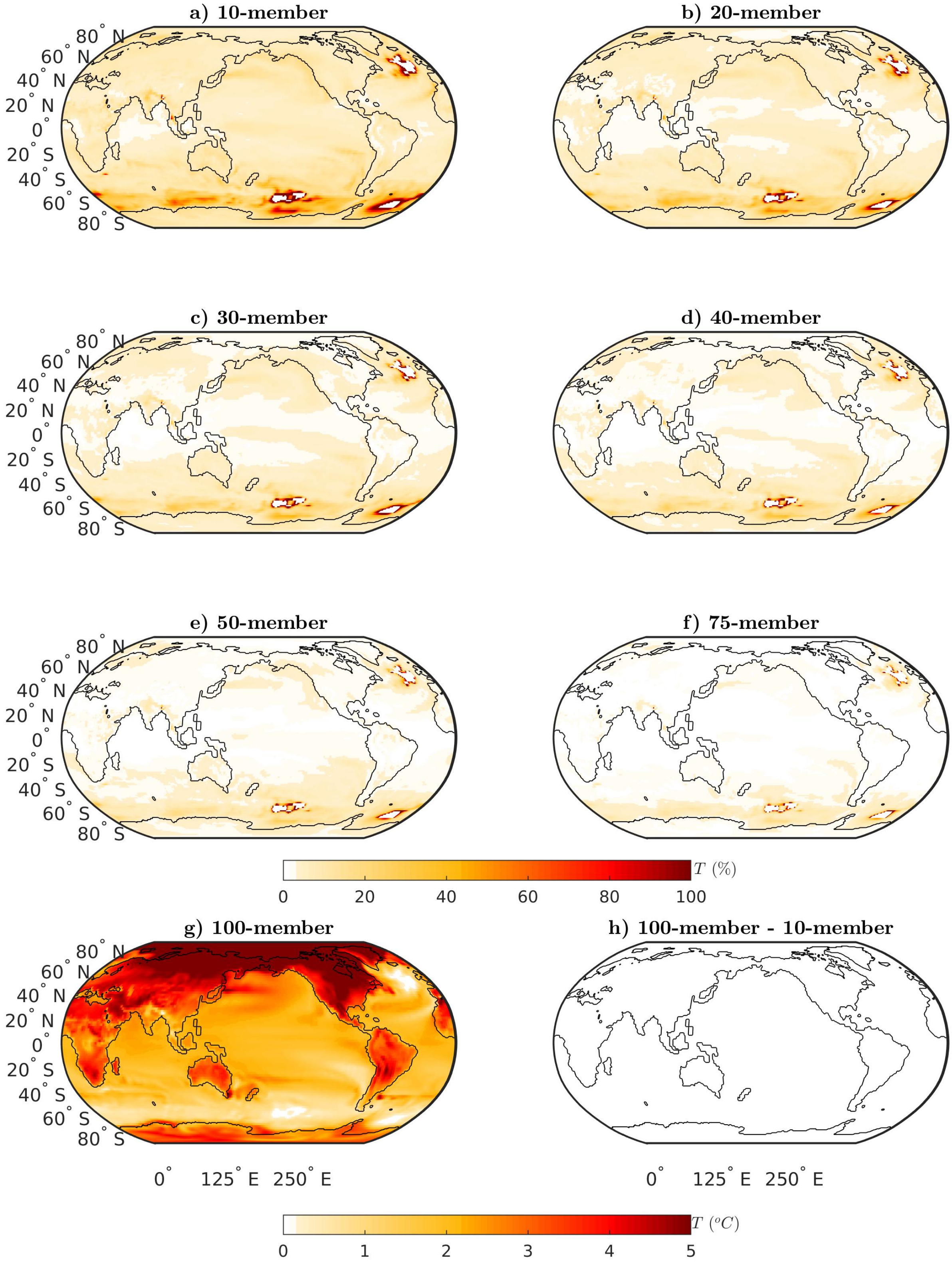

Supplementary Figure 1: **Role of ensemble size in causing uncertainty in the forced response in temperature in a individual single model initial-condition large ensemble (SMILE) ($\Delta T_{s,\mathbf{F}}$).** Computed for 100 re-samples (no replacement) of MPI-GE. Shown is the difference between the maximum value of $\Delta T_{s,\mathrm{F}}$ and the minimum value for 10, 20, 30, 40, 50 and 75 members shown as a percentage of the 100-member estimate. The 100-member estimate of the forced response is shown in the bottom left panel. The difference between the 100-member estimate and the mean of 100 20-member estimates is shown in the bottom right panel.

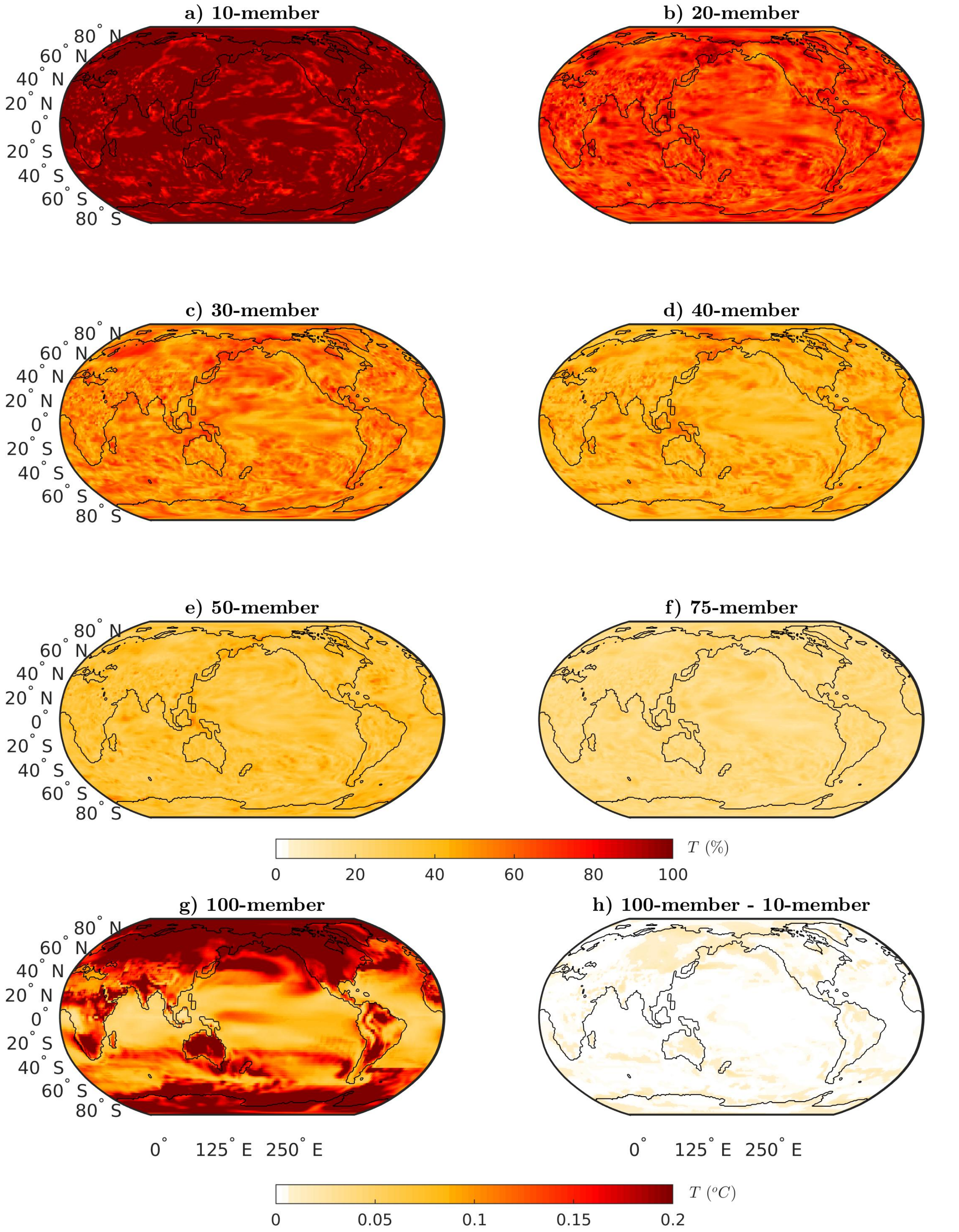

Supplementary Figure 2: **Role of ensemble size in causing uncertainty in the internal variability of projections of temperature in a individual single model initial-condition large ensemble (SMILE) ($\sigma(\Delta T_s)$).** Computed for 100 re-samples (no replacement) of MPI-GE. Shown is the difference between the maximum value of $\sigma(\Delta T_s)$ and the minimum value for 10, 20, 30, 40, 50 and 75 members shown as a percentage of the 100-member estimate. The 100-member estimate of the forced response is shown in the bottom left panel. The difference between the 100-member estimate and the mean of 100 20-member estimates is shown in the bottom right panel.

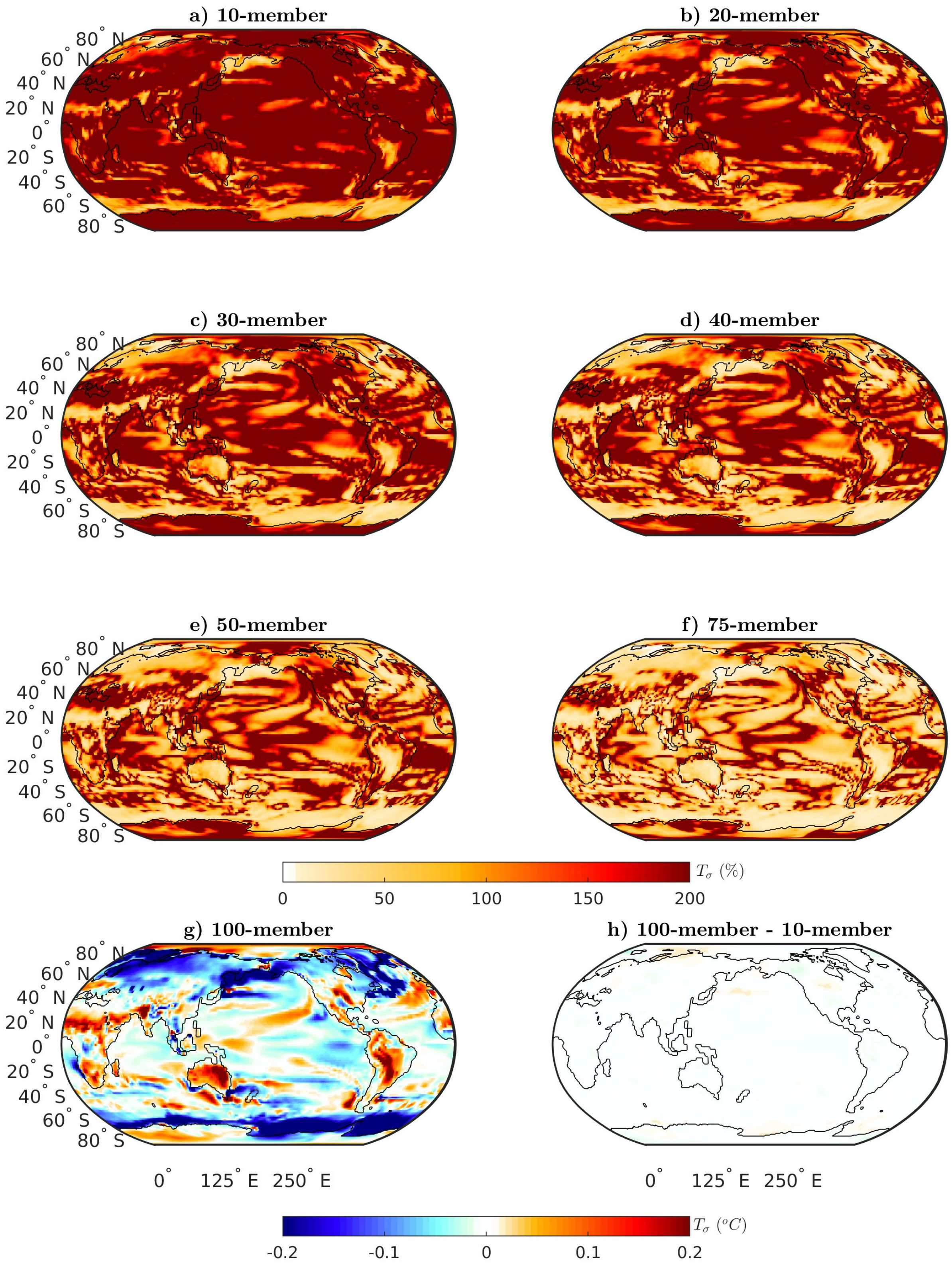

Supplementary Figure 3: **Role of ensemble size in causing uncertainty in the forced response in temporal temperature variability in a individual single model initial-condition large ensemble (SMILE) ($\Delta T_{\sigma,s,\mathbf{F}}$)** Computed for 100 re-samples (no replacement) of MPI-GE. Shown is the difference between the maximum value of $\Delta T_{\sigma,s,\mathrm{F}}$ and the minimum value for 10, 20, 30, 40, 50 and 75 members shown as a percentage of the absolute value of the 100-member estimate. The 100-member estimate of the forced response is shown in the bottom left panel. The difference between the 100-member estimate and the mean of 100 20-member estimates is shown in the bottom right panel.

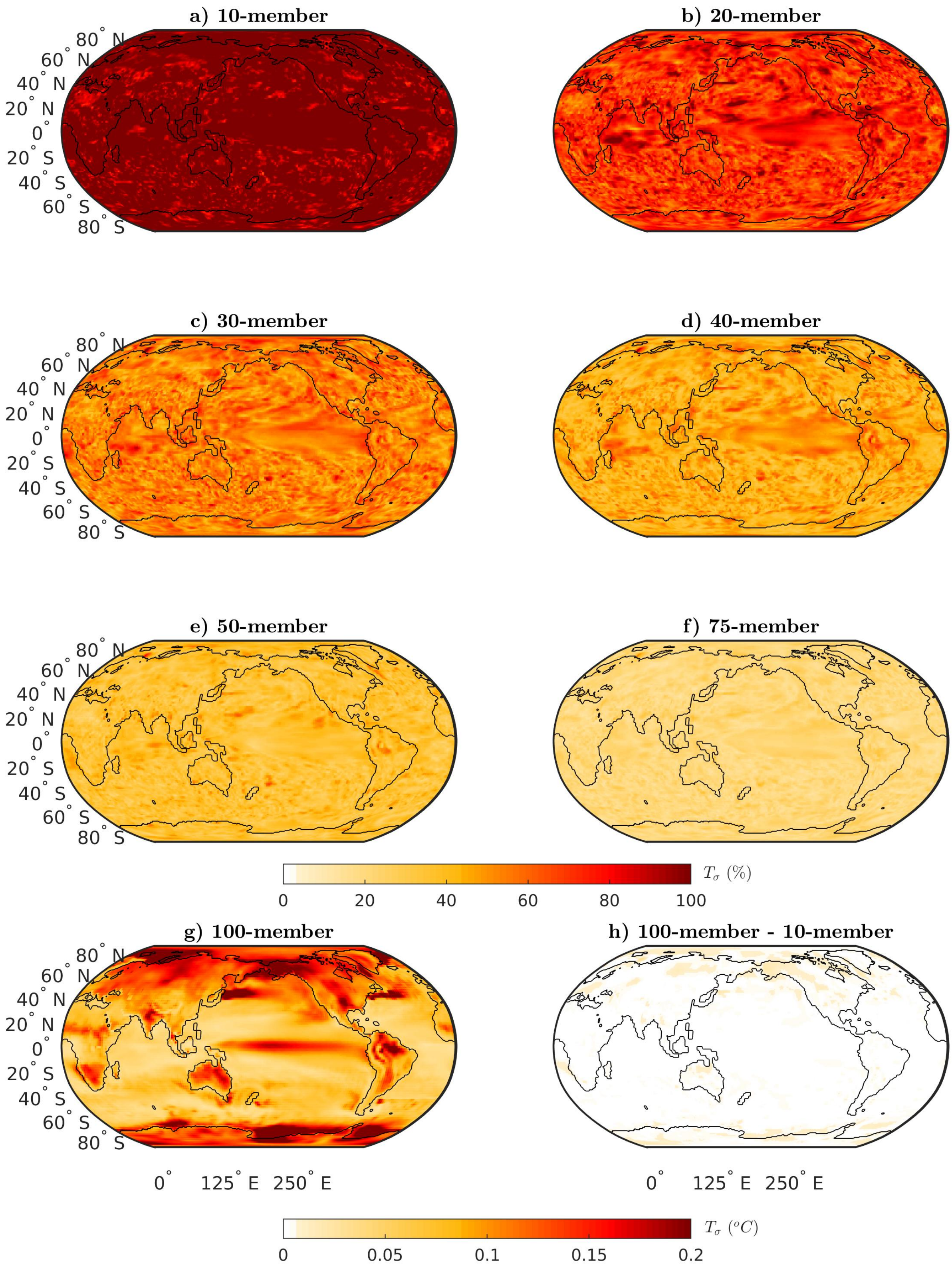

Supplementary Figure 4: **Role of ensemble size in causing uncertainty in the internal variability of projections of temporal temperature variability in a individual single model initial-condition large ensemble (SMILE) ($\sigma(\Delta T_{\sigma,s})$).** Computed for 100 re-samples (no replacement) of MPI-GE. Shown is the difference between the maximum value of $\sigma(\Delta T_{\sigma,s})$ and the minimum value for 10, 20, 30, 40, 50 and 75 members shown as a percentage of the 100-member estimate. The 100-member estimate of the forced response is shown in the bottom left panel. The difference between the 100-member estimate and the mean of 100 20-member estimates is shown in the bottom right panel.

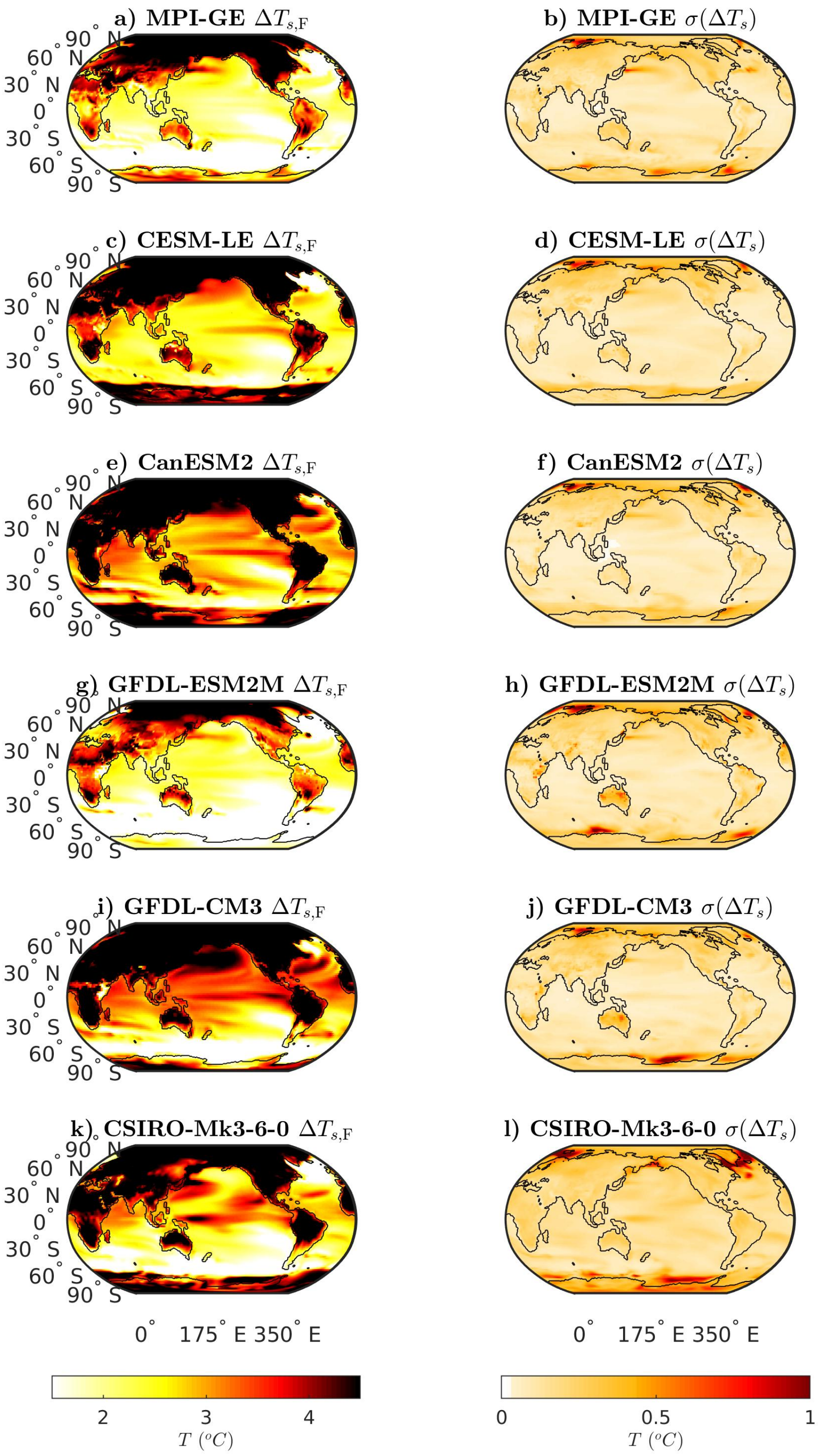

Supplementary Figure 5: **Forced response and internal variability of the forced response in temperature ($\Delta T_{s,\mathbf{F}}$ & $\sigma(\Delta T_s)$) for each single model initial-condition large ensemble (SMILE).** a,b) MPI-GE, c,d) CESM-LE, e,f) CanESM2, g,h) GFDL-ESM2M, i,j) GFDL-CM3 and k,l) CSIRO-Mk3-6-0

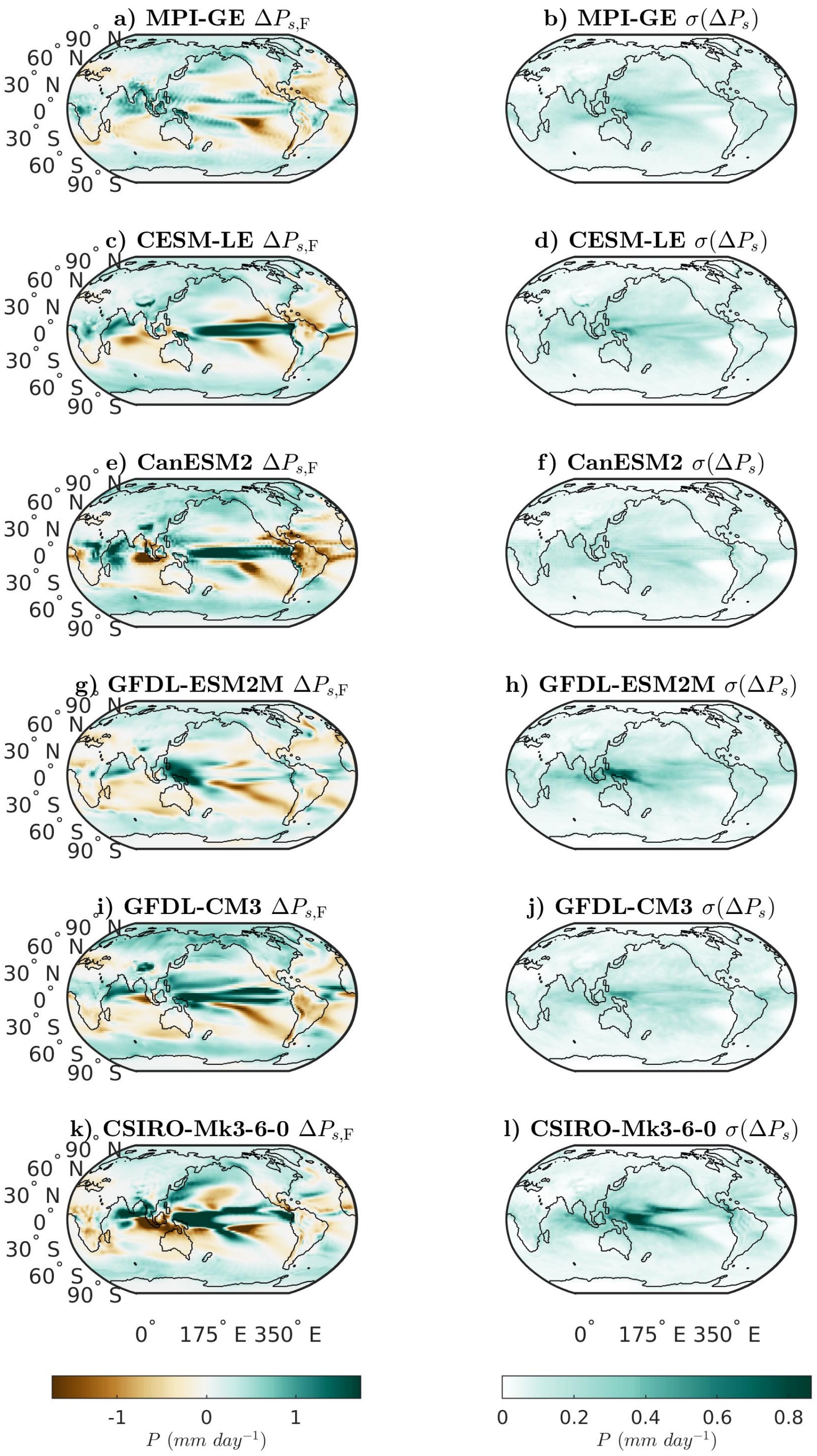

Supplementary Figure 6: **Forced response and internal variability of the forced response in precipitation ($\Delta P_{s,\mathbf{F}}$ & $\sigma(\Delta P_s)$) for each single model initial-condition large ensemble (SMILE).** a,b) MPI-GE, c,d) CESM-LE, e,f) CanESM2, g,h) GFDL-ESM2M, i,j) GFDL-CM3 and k,l) CSIRO-Mk3-6-0

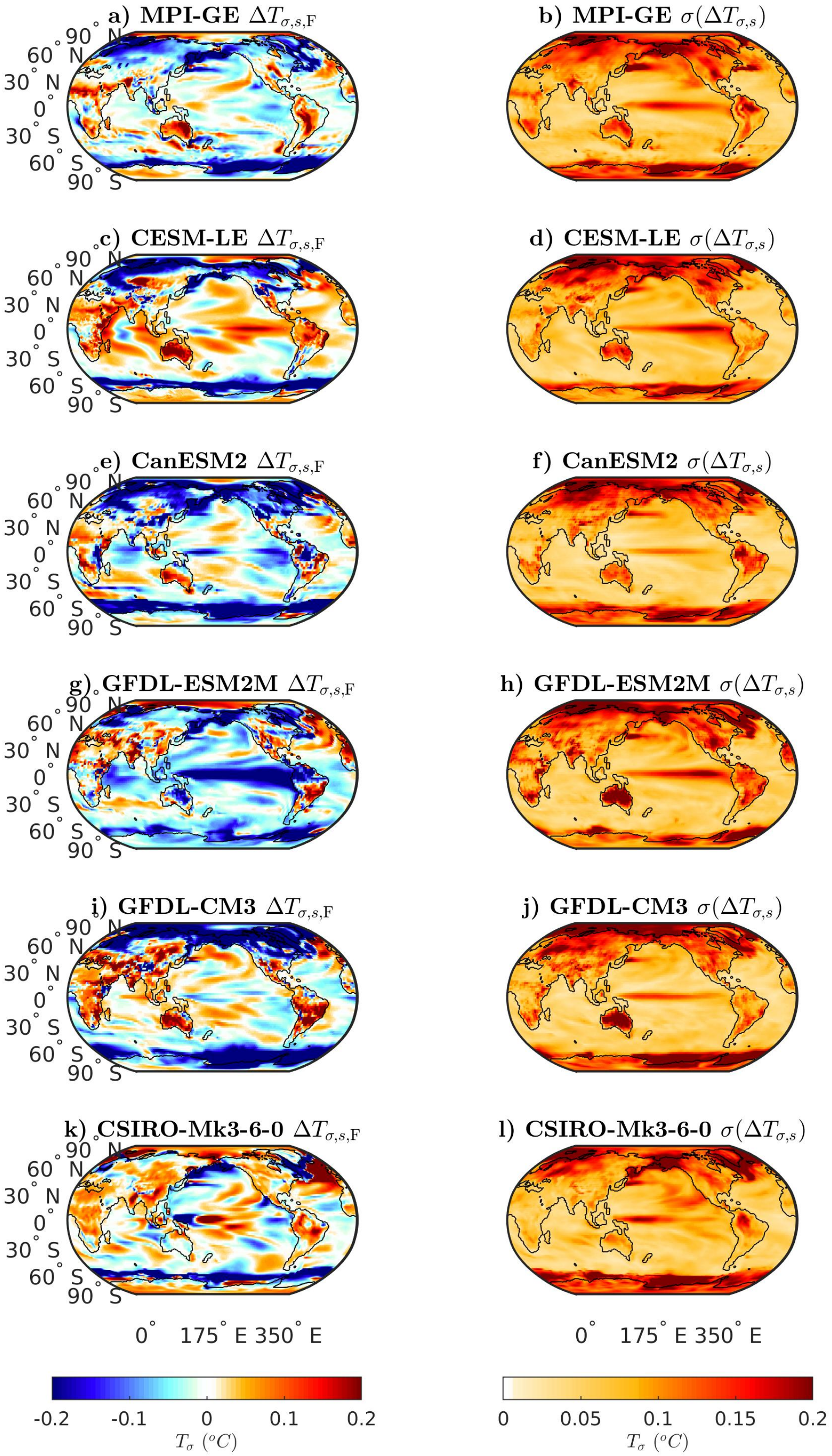

Supplementary Figure 7: **Forced response and internal variability of the forced response in temporal temperature variability ($\Delta T_{\sigma,s,\mathbf{F}}$ & $\sigma(\Delta T_{\sigma,s})$) for each single model initial-condition large ensemble (SMILE).** a,b) MPI-GE, c,d) CESM-LE, e,f) CanESM2, g,h) GFDL-ESM2M, i,j) GFDL-CM3 and k,l) CSIRO-Mk3-6-0

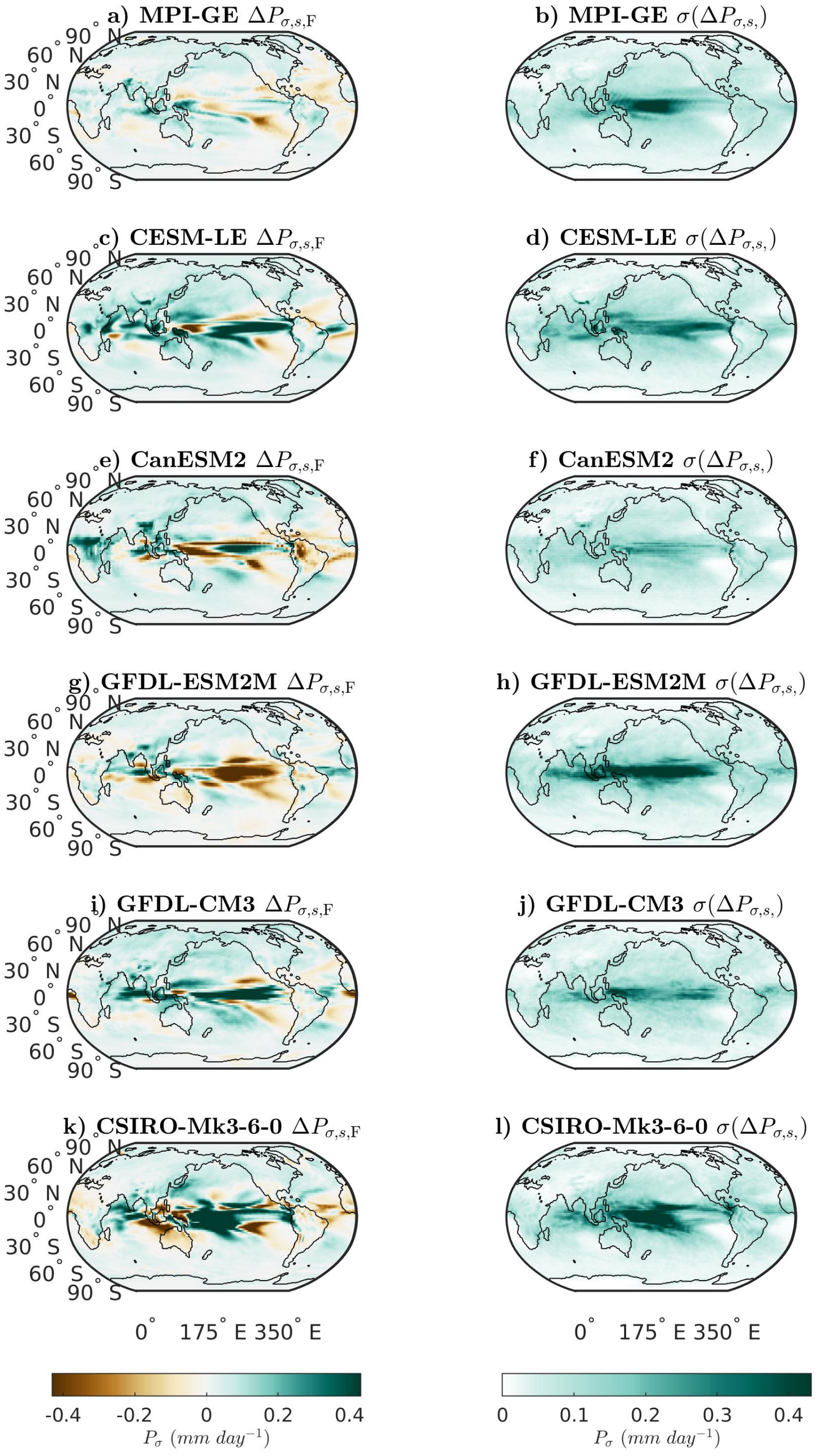

Supplementary Figure 8: **Forced response and internal variability of the forced response in temporal precipitation variability ($\Delta P_{\sigma,s,\mathbf{F}}$ & $\sigma(\Delta P_{\sigma,s})$) for each single model initial-condition large ensemble (SMILE).** a,b) MPI-GE, c,d) CESM-LE, e,f) CanESM2, g,h) GFDL-ESM2M, i,j) GFDL-CM3 and k,l) CSIRO-Mk3-6-0

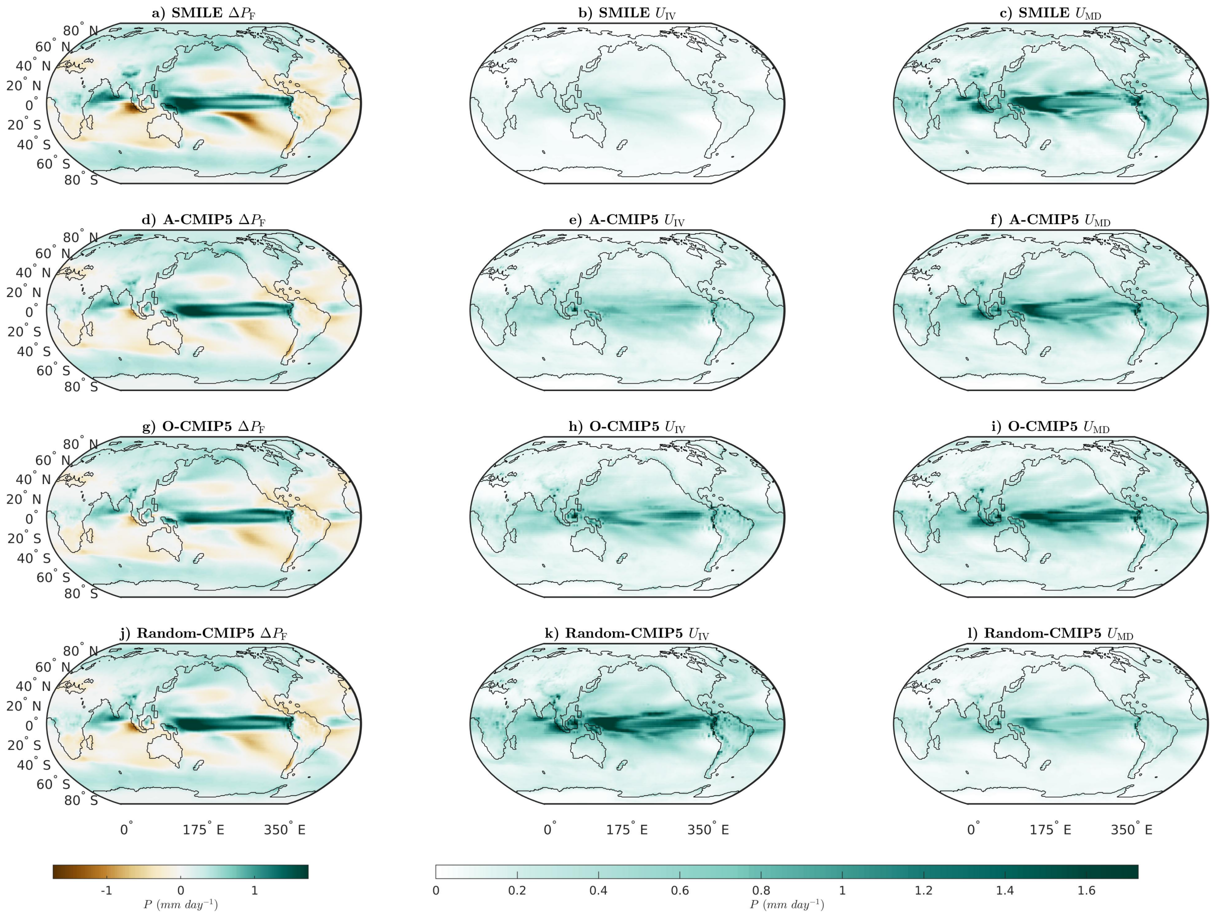

Supplementary Figure 9: **Long-term projections of the mean-state precipitation response to external forcing (ΔP) and the contribution of the uncertainty due to internal variability ($U_{\mathbf{IV}}$) and model-to-model differences ($U_{\mathbf{MD}}$).** Shown for a,b,c) Six single model initial-condition large ensembles (SMILEs), d,e,f) CMIP5 sub-ensembles that share the atmosphere component (A-CMIP5), g,h,i,) CMIP5 sub-ensembles that share the ocean component (O-CMIP5), j,k,l) CMIP5 randomly subset into ensembles (Random-CMIP5), this random sampling is completed 30 times and averaged.

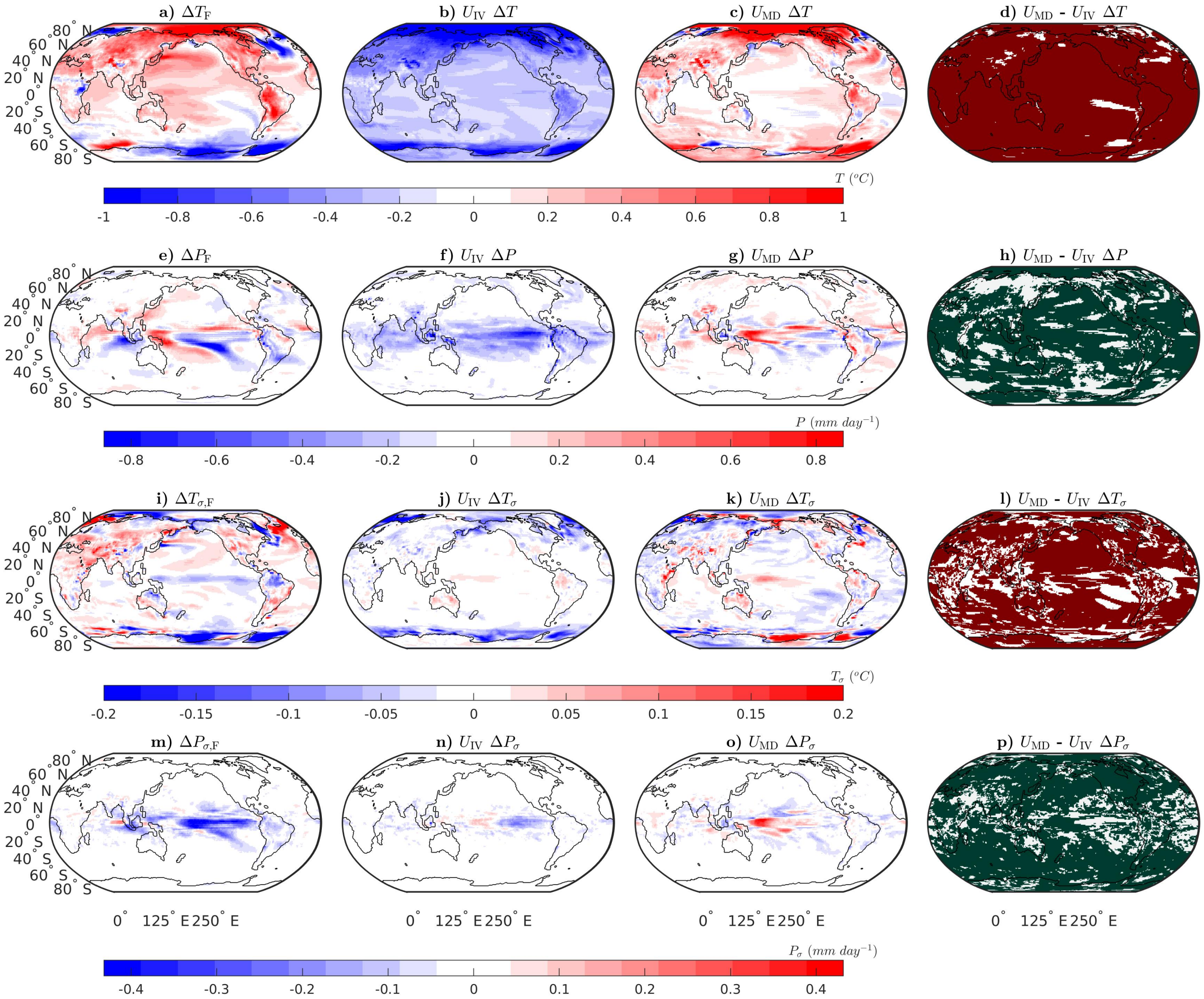

Supplementary Figure 10: **Difference between the single model initial-condition large ensemble (SMILE) and CMIP5 sub-ensemble methodologies.** SMILE minus CMIP5 atmospheric sub-ensembles for left to right: forced response (Δ_{F}), uncertainty due to internal variability (U_{IV}), uncertainty due to model-to-model differences (U_{MD}), and U_{MD} minus U_{IV}. Shown for projections of a-d) temperature (ΔT), e-h) precipitation (ΔP), i-l) temporal temperature variability (ΔT_{σ}), and m-p) temporal precipitation variability (ΔP_{σ}). Far right panels are red (temperature) or green (precipitation) when the CMIP5 atmospheric sub-ensembles and SMILE analyses agree on whether U_{IV} or U_{MD} is larger.

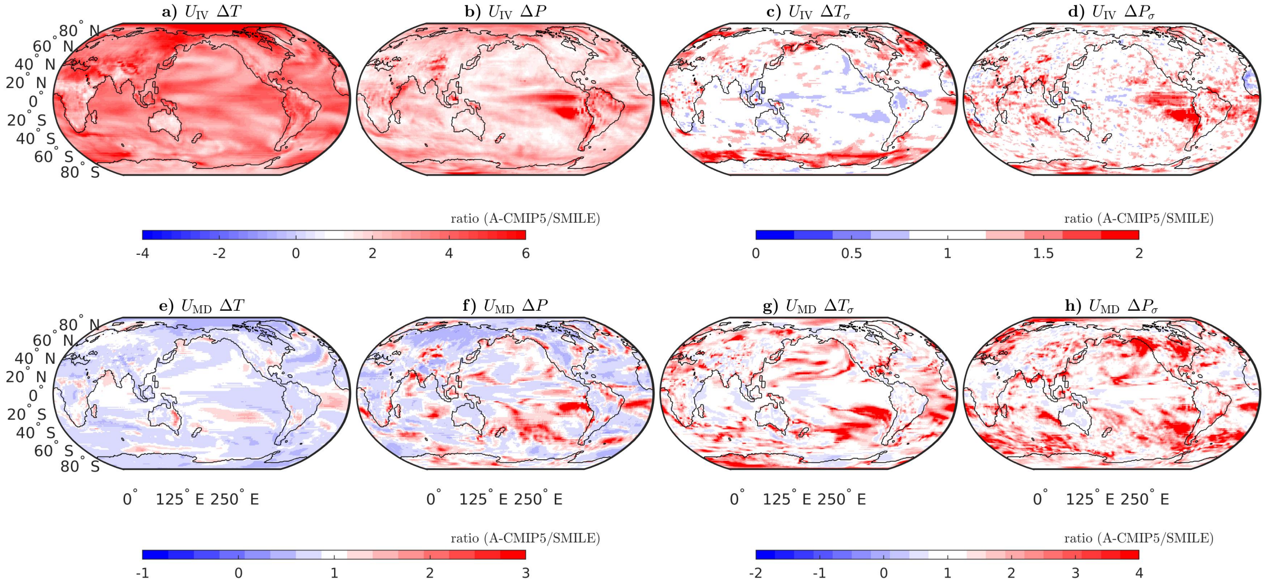

Supplementary Figure 11: **Ratio between the CMIP5 atmospheric sub-ensembles and single model initial-condition large ensemble (SMILE) results** Top row: uncertainty due to internal variability (U_{IV}) for the CMIP5 atmospheric sub-ensembles divided by U_{IV} from the SMILE analysis. Bottom row: uncertainty due to model-to-model differences (U_{MD}) for the CMIP5 atmospheric sub-ensembles divided by U_{MD} from the SMILE analysis. Shown for projections of a,e) temperature (ΔT), b,f) precipitation (ΔP), c,g) temporal temperature variability (ΔT_σ), and d,h) temporal precipitation variability (ΔP_σ).

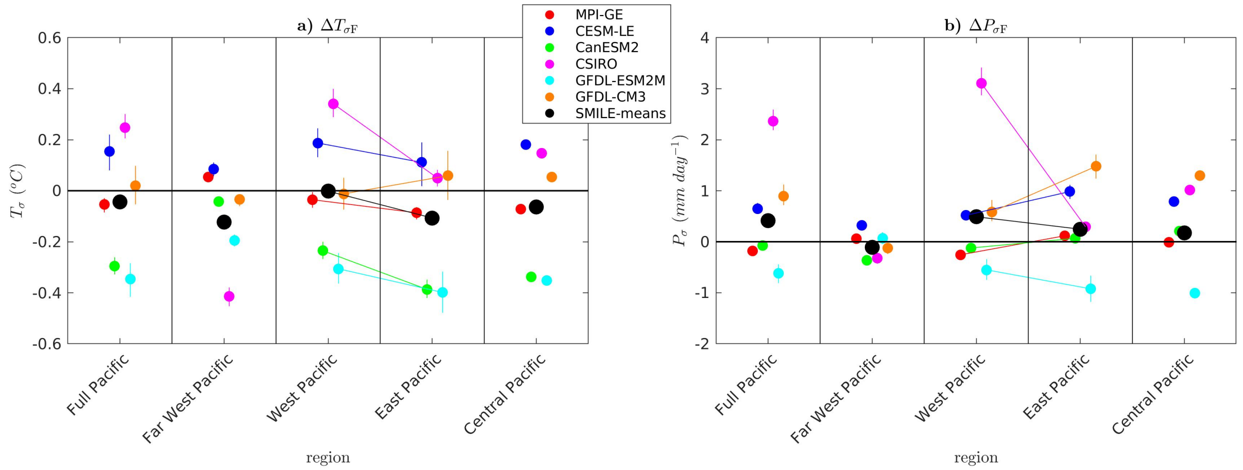

Supplementary Figure 12: **Projections of externally forced responses in temporal variability (temperature; T_σ & precipitation; P_σ) in the tropical Pacific in austral summer**. Forced response over the full ($160^oE - 260^oE$), east ($220^oE - 260^oE$), central ($190^oE - 240^oE$), west ($160^oE - 220^oE$) and far west ($120^oE - 160^oE$ Pacific (all between $5^oS - 5^oN$) in each individual single model initial-condition large ensemble (SMILE) ($\Delta_{s,\mathrm{F}}$; coloured circles) and the SMILE mean (Δ_{F}; black circle) are shown for a) T_σ and b) P_σ. Calculations are completed on the austral summer mean (December, January, February). Horizontal lines are shown between the east and west Pacific to illustrate the proportional change in each variable. Errorbars are computed by bootstrapping 1000 times with the matlab *bootci* function for the mean of equation 10 (see methods).

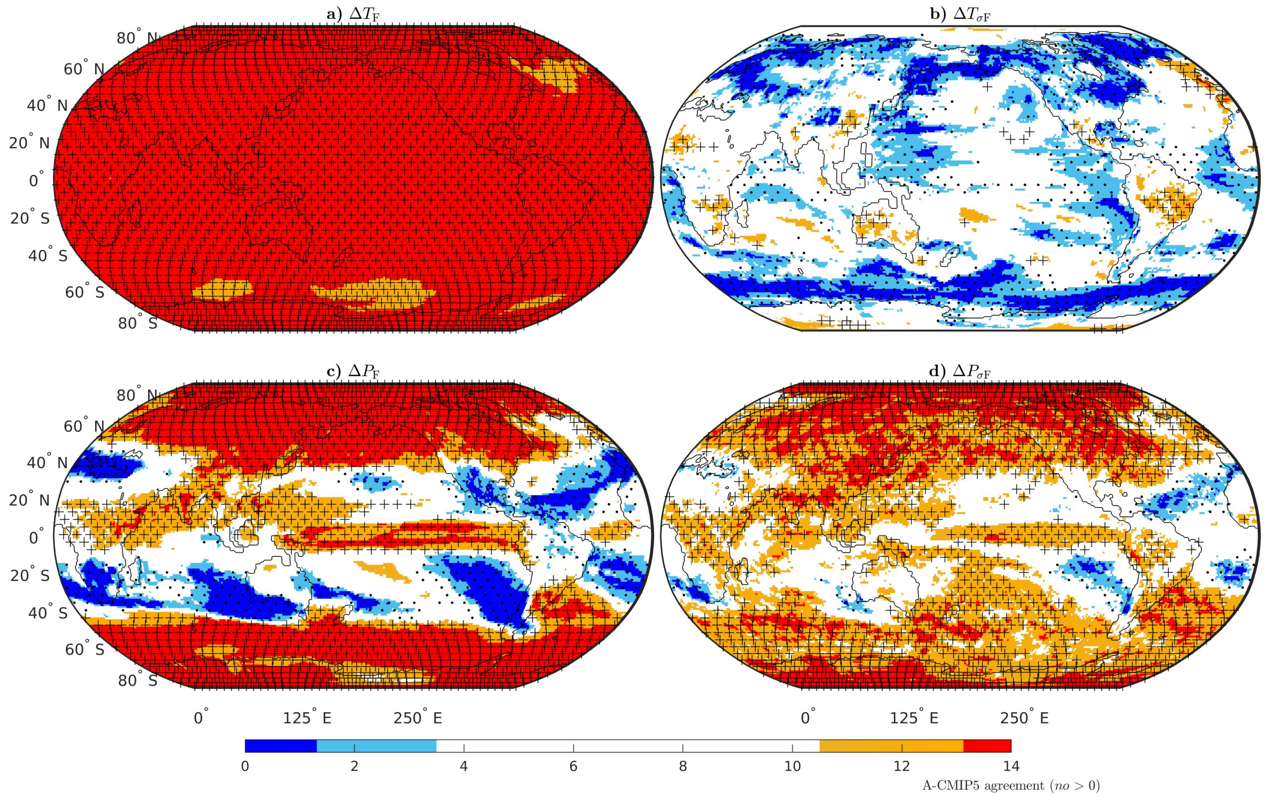

Supplementary Figure 13: **Model-to-model agreement on the sign of the forced response to external forcing using the CMIP5 multi-model ensemble.** a) mean-state temperature (ΔT_{F}), b) temporal temperature variability ($\Delta T_{\sigma \mathrm{F}}$), c) mean-state precipitation (ΔP_{F}) and d) temporal precipitation variability ($\Delta P_{\sigma \mathrm{F}}$). The colours show agreement for the CMIP5 atmospheric sub-ensembles. Red shows agreement in an increase in each quantity while blue shows agreement in a decrease. White regions have less than 79% agreement on the sign of the change (less than 11 of 14 sub-ensembles agree). Stippling shows where there is 68% agreement on the sign of the change using CMIP5 r1i1p1 ensemble members (25 or more out of the 37 subsets agree), with crosses indicating an increase and dots indicating a decrease. The measures of agreement correspond to a significance level of 0.01 using a binomial distribution.

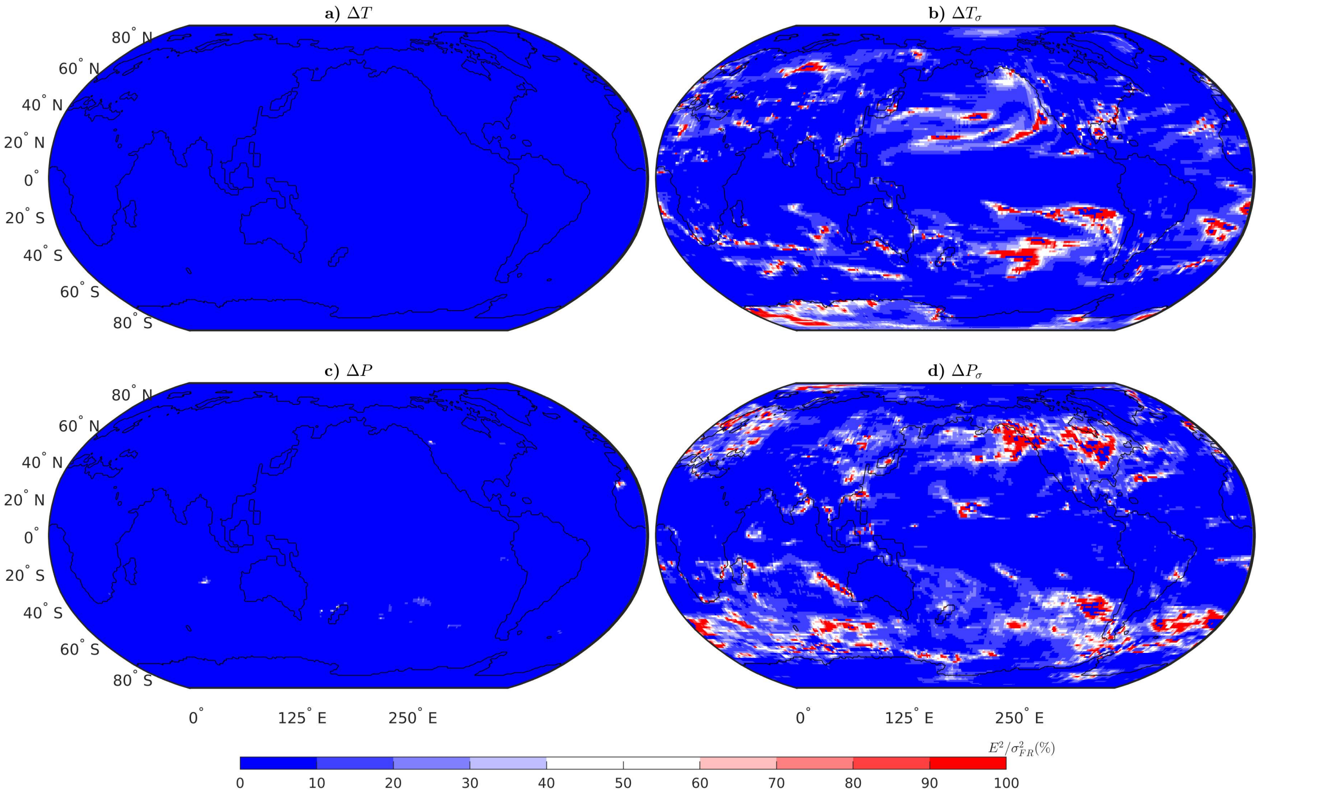

Supplementary Figure 14: **Relative magnitude of the terms E^2 compared to σ^2_{FR} from equation 6 of the main paper**. This term is used to correct the biased estimate of the uncertainty due to model-to-model differences (U_{MD}) due to the fact the the ensemble is not infinite in size [9]. Shown for projections of a) temperature (ΔT), b) temporal temperature variability (ΔT_σ), c) precipitation (ΔP), and d) temporal precipitation variability (ΔP_σ)

Supplementary References

[1] C. Deser et al. Strength in Numbers: The Utility of Large Ensembles with Multiple Earth System Models. *Nature Climate Change*, 2020. doi: https://doi.org/10.1038/s41558-020-0731-2.

[2] N. Maher et al. The Max Planck Institute Grand Ensemble: Enabling the Exploration of Climate System Variability. *Journal of Advances in Modeling Earth Systems*, 11(7):2050–2069, 2019. doi: 10.1029/2019MS001639.

[3] J. E. Kay et al. The Community Earth System Model (CESM) Large Ensemble Project: A Community Resource for Studying Climate Change in the Presence of Internal Climate Variability. *Bulletin of American Meteorological Society*, 96(8):1333–1349, 2015. ISSN 0003-0007. doi: 10.1175/BAMS-D-13-00255.1.

[4] M.C. Kirchmeier-Young, F.W. Zwiers, and N.P. Gillett. Attribution of Extreme Events in Arctic Sea Ice Extent. *Journal of Climate*, 30(2):553–571, 2017. doi: 10.1175/JCLI-D-16-0412.1.

[5] K. B. Rodgers, J. Lin, and T. L. Frölicher. Emergence of multiple ocean ecosystem drivers in a large ensemble suite with an Earth system model. *Biogeosciences*, 12(11):3301–3320, 2015.

[6] L. Sun, M. Alexander, and C. Deser. Evolution of the Global Coupled Climate Response to Arctic Sea Ice Loss during 1990–2090 and Its Contribution to Climate Change. *Journal of Climate*, 31(19):7823–7843, 2018. doi: 10.1175/JCLI-D-18-0134.1.

[7] S.J. Jeffrey et al. Australia's CMIP5 submission using the CSIRO-Mk3.6 model. *Australian Meteorological and Oceanographic Journal*, 63(1):1–13, 2012.

[8] J. Boé. Interdependency in multimodel climate projections: Component replication and result similarity. *Geophysical Research Letters*, 45(6):2771–2779, 2018. doi: 10.1002/2017GL076829.

[9] D.P. Rowell, C.K. Follans, K. Maskell, and M.N. Ward. Variability of summer rainfall over tropical north Africa (1906-92): Observations and modelling. *Quarterly Journal of the Royal Meteorological Society*, 121:669–704, 1995.
